# Socioeconomic status as a moderator between frailty and mortality at old ages

**DOI:** 10.1186/s12877-016-0322-2

**Published:** 2016-08-09

**Authors:** Danan Gu, Fang Yang, Jessica Sautter

**Affiliations:** 1 United Nations Population Division, Two UN Plaza, DC2-1910, New York, NY 20012 USA; 2 Department of Social Work, School of Sociology and Political Science, Shanghai University, Shanghai, China; 3 Department of Behavioral and Social Sciences, University of the Sciences, Philadelphia, USA

**Keywords:** Frailty, Frailty index, Mortality, Socioeconomic status, Socioeconomic vulnerability, Older adults, China, Moderator

## Abstract

**Background:**

Despite the well-established power of frailty to predict mortality, and the known associations of socioeconomic status (SES) with mortality, it is largely unknown whether the linkage between frailty and mortality varies across different SES groups. This study aims to investigate whether SES moderates the association between frailty and mortality.

**Methods:**

We relied on the 2008/2009 and 2011/2012 waves of the Chinese Longitudinal Healthy Longevity Survey, a nationwide sample of 13,731 adults aged 65 or older in China. Frailty was constructed using a cumulative index of 38 items (with 39 deficits) reflecting different dimensions of health; the index or the proportion of deficits ranges from 0 to 1, with greater scores indicating poorer health condition. SES was measured by a socioeconomic vulnerability index (SEVI) also from a similar cumulative approach consisting of 6 deficits; the proportion of deficits ranges from 0 to 1 with higher scores indicating lower SES. Eight Weibull hazard regression models were performed to examine how SES moderates the linkage between frailty and mortality.

**Results:**

We found that a one percentage point increase in the frailty index was associated with an increased hazard ratio (HR) by 2.7 % (HR = 1.027, 95 % CI: 1.025–1.027); a one percentage point increase in SEVI score was associated with an increased hazard ratio by 0.6 % (HR = 1.006, 95 % CI: 1.004–1.008) controlling for demographics. When interactions between SEVI and frailty index were modeled, the increased mortality risk associated with frailty was weaker among people with lower SES than among people with higher SES (HR = 0.983, 95 % CI: 0.967–0.992). However, the moderating role of SES was diminished when interactions between SES and age and between frailty and age were modeled. With increasing age, the increased mortality risks associated with frailty and socioeconomic vulnerability weakened.

**Conclusions:**

Frailty was a stronger predictor of mortality among individuals with higher SES than those with lower SES. The increased mortality risks associated with socioeconomic vulnerability and frailty weakened with age. Public health programs aimed at improving SES and promoting healthy longevity should start early in old age, or even earlier, and target poor and frail older adults for maximum impact.

## Background

Frailty is a physiological state marked by dysregulation in multiple bodily systems and increased vulnerability to adverse outcomes [1–6]. Among the various ways to operationalize frailty, the frailty index is based on the widely used deficit accumulation approach [7–16]. The frailty index incorporates a broad range of psychological, physiological, and functional variables to represent the proportion of health deficits an individual currently has to the total number of possible deficits; this is a good proxy for biological aging [2, 3, 5, 6, 17], mortality prediction [6, 10–12, 18, 19], falls and hospitalizations [7–9, 16–21], and overall health condition [2, 3, 13–16]. The significant predictive power of frailty on mortality and health outcomes persists across different populations and cultural contexts in both Western and non-Western societies [6, 10–12, 17–19]. Several studies further examined the predictive power of frailty on mortality at different age groups [19–21]; frailty in terms of cumulative deficits is still a robust predictor of subsequent mortality at oldest-old ages, even in centenarians, although the predictive power was weakened in centenarians as compared to other old age groups [19, 20].

Additional empirical studies in sociology and social gerontology have shown that socioeconomic status (SES) plays an important role in health and mortality at old ages because a high SES provides older adults with material resources, helps them develop healthy lifestyles, and confers psychological benefits; consequently, older adults with a higher SES tend to have a lower likelihood of mortality than their lower SES counterparts [22–32]. A branch of studies have further investigated the role of SES, measured by education and/or income, as a mediator or moderator for the relationship between self-rated health and mortality in general adult populations [33–40], yet studies with other health indicators are rare. Among the studies focusing on the mediating or moderating role of SES in the linkage between self-rated health and mortality, the findings were divided and the conclusions were mixed. While some found no significant mediating role [39–41], others found a significant mediating or moderating role of SES [33–38]. Some found that self-rated health was more strongly associated with mortality for adults with higher education and/or income relative to their lower SES counterparts [33–35, 38], whereas two studies found just the opposite [36, 37]. However, among all these studies, only one focused on older adults [35].

To date, despite the well-established power of frailty to predict mortality, and the known associations of SES with mortality, it is largely unknown whether the linkage between frailty and mortality varies across different SES groups. We have not found any studies in developing or developed countries that examine this research question. To address the gap in the literature, we aim to investigate whether SES modulates the predictive power of frailty on mortality using a large nationally representative sample of older adults in mainland China with more than 13,700 adults aged 65 or older.

## Methods

### Study sample

We used the 2008/2009 and 2011/2012 waves of the nationally representative Chinese Longitudinal Healthy Longevity Survey (CLHLS) to fulfill our research goals. Started in 1998, each wave of the CLHLS sampled half of the counties/cities in 22 Han-ethnicity dominant provinces out of 31 provinces in mainland China, accounting for 82 % of the total population in China in 2010. One aim of the CLHLS is to interview all centenarians in the sampled counties/cities. Age validation of each centenarian in the CLHLS is comprehensive, including validations from birth certificates, genealogical documents, household booklets, and ages of their children and siblings whenever available. For each centenarian sampled, roughly one nearby respondent with predesignated age and sex from each of three age groups (65–79, 80–89, and 90–99) was randomly chosen to be interviewed. The term "nearby" could refer to neighboring villages or towns, depending on availability of persons with predesignated age and sex. All information was obtained through in-home interviews.

Detailed sampling procedures can be found elsewhere [42]. According to relevant publications [42], the accuracy of age reporting; the randomness of attrition; and the reliability, validity, and consistency of numerous measures in the CLHLS is high for all waves. Approximately 17 % of the 16,563 respondents interviewed in 2008/2009 were lost to follow-up in the 2011/2012 wave. Loss to follow-up is mainly due to resettlement and frequent changes in the administrative boundaries of counties and/or districts related to urbanization [42]. We excluded participants lost to follow-up due to their unknown survival status. This leaves a valid sample size of 13,731 for the analyses; 39.7 % died before the 2011/2012 wave and the remaining 61.3 % survived to the 2011/2012 interview. Compared to the analytic sample, they were more likely to have poorer health and high socioeconomic status, to be unmarried, women, Han ethnicity, non-smokers, and to not coreside with family members.

### Measurements

#### Mortality

Mortality risk was the dependent variable in survival analyses, measured with survival status (died or survived in the 2011/2012 wave) and the duration of survival (days lived between the date of the 2008/2009 interview and the date of the 2011/2012 interview or death). For those who died before the 2011/2012 interview, date of death was collected from officially issued death certificates whenever available (more than 80 % cases); next-of-kin and local residential committees were consulted when a death certificate was not available. Our supplementary analysis showed that the data quality of death rates in the CLHLS from 2008/2009 to 2011/2012 was comparable to estimates derived from the censuses for ages 95 years and younger and had a better quality than census estimates for ages 96 +.

#### Frailty

Following an established method [19], we used a cumulative approach to construct a frailty index based on 38 items reflecting different dimensions of health, including cognitive function, functional limitations, activities of daily living, instrumental activities of daily living, chronic disease conditions, and so forth. Each individual item was coded as 1 if a deficit was present and 0 otherwise. Following other studies in the literature [19, 21], we assigned a score of 2 if the respondent had a serious illness that caused the respondent to be hospitalized or bedridden two or more times in the past two years. Each respondent's deficit score was then obtained by summing the number of cumulative deficits (0–39) and dividing the number of deficits by the possible total (i.e., 39) to obtain a frailty index proportion value from 0 to 1. The validity of the frailty index constructed using this approach with CLHLS data has been verified in previous studies [19, 43]. Appendix 1 provides a comparison between the age-sex-specific distribution of the frailty index in the 2008/2009 CLHLS and that in the previous literature. Appendix 2 further compares mortality rate by frailty index score between the CLHLS 2008/2009-2011/2012 and the previous studies based on other datasets. The comparisons clearly indicate that the frailty index in the CLHLS is valid. This cumulative approach to constructing the frailty index best captures the overall health reserve of an individual [13, 15, 16].

#### Socioeconomic Status (SES)

Following a similar cumulative approach to a social vulnerability index in the literature [11], we constructed a SES vulnerability index with higher score indicating poor SES (thereafter socioeconomic vulnerability index, SEVI). The SEVI consists of 6 variables: educational attainment, primary lifetime occupation, economic independence, family economic status, access to healthcare services, and urban-rural residence [19, 33, 34]. The inclusion of urban-rural residence as a proxy measure of SES in China is a common practice in the literature because urban and rural areas are greatly different in terms of socioeconomic development level and social welfare system due to the unique dual socioeconomic system between these two areas in China [25, 26]. Each SES component was scored as follows: for educational attainment, 0 years of schooling = 1, 1–6 years of schooling = 0.5, and 7+ years of schooling = 0; for primary lifetime occupation, white collar = 0 and other types = 1; for economic independence, daily expenses mainly covered by one’s own work or retirement pension/wage = 0, otherwise 1; for family economic status, very rich = 0, rich = 0.25, so so = 0.5, poor = 0.75, and very poor = 1; for access to healthcare services, timely access = 0, otherwise 1; and for urban-rural residence, city = 0, town = 0.5, and rural = 1. The value of the SEVI for each respondent was obtained by summing all values over these six variables (0–6, with higher scores denoting lower levels of SES). Dividing the socioeconomic vulnerability score by 6, we obtained the proportional SEVI score, ranging from 0 to 1. The reliability coefficient of SEVI is 0.62.

#### Covariates

Because previous empirical studies have shown that demographics, family/social support, and health practices are all associated with frailty and mortality in older adults [19, 33, 34], we included the following dichotomous covariates to obtain robust results: age (single year), sex (man vs. woman), ethnicity (Han vs. non-Han), marital status (currently married vs. not married), coresidence with family (yes vs. no), current smoking (yes vs. no), and regular exercise (yes vs. no).

### Analytical strategies

To examine the relationship between frailty and mortality, how the relationship varies by SES, and how the SES moderating role is altered when other factors are present, we employed eight Weibull hazard regression models of survival analysis. Model I included frailty index and SEVI, controlling for age, sex, and ethnicity. Model II added a two-way interaction between frailty index and SEVI, and Model III further added other controls (coresidence with family, current marital status, current smoking status, and whether the respondent does regular exercise) to Model II. Model IV added an interaction between SEVI and age to Model II, and Model V added an interaction between frailty index and age to Model II. Model VI added remaining controls to Model V. Model VII was designed to add interactions between SEVI and age and between frailty index and age in Model II, and Model VIII was designed to include remaining controls for Model VII. We tested a three-way interaction between frailty, SES, and age but it was not significant. We also did not include interactions with sex because all interactions between sex, SES, and frailty were not significant.

In survival analysis, the length of survival time for survivors was calculated as number of days between the date of the 2008/2009 interview and the date of the 2011/2012 interview; for deceased respondents, survival time was the number of days between the date of the 2008/2009 interview and the date of death. We excluded those who were lost to follow up in the 2011/2012 survey; an alternative approach that imputed survival status by assuming that respondents lost to follow-up had the same survival status and length of survival as respondents with the same demographics, SES, family/social support, health practice, and health conditions in 2008/2009 produced similar results to those reported here [Appendix 3].

The proportion of missing values for all variables in the analysis was less than 2 %. To reduce possible bias due to missing values, we employed multiple imputation for all variables. Other alternative approaches such as mean, mode, or median imputation were tested and produced almost identical results. We did not apply sampling weights in the regression models because the CLHLS weight variable was unable to reflect the national population distributions with respect to variables other than age, sex, and urban/rural residence [44]. All analyses were performed using STATA 13.0.

## Results

Table 1 presents a description of the whole sample aged 65 or older. About 40 % of the respondents died before the 2011/2012 interview. The mean frailty index score was 0.21 for the entire sample, with an average of 8 deficits; mean socioeconomic vulnerability index score was 0.63, with an average of 3.77 deficits. Because the CLHLS oversampled respondents with an older age, the proportions of centenarians and nonagenarians in the sample were very high. About 57 % of participants were women, 93 % were Han ethnicity, 83 % resided with their families, 30 % were currently married, 18 % were current smokers, and 27 % regularly exercised. Death, frailty, and SEVI varied across categories for almost all covariates. For example, older individuals had higher proportions of death, higher frailty index scores, and higher SEVI scores. The lower frailty index score for smokers is possibly because of their younger average age (83 years) compared to non-smokers (88 years).

**Table 1:** Sample description and patterns in mortality, frailty index, and socioeconomic vulnerability index CLHLS 2008/2009-2011/2012

	%	% died	Mean score of the frailty index ^a^	Mean score of the socioeconomic vulnerability index ^b^
Total (*n* = 13,731)	100.0	39.7	0.21 (8.06)	0.63 (3.77)
Control variables				
Ages 65–79	26.9	10.7	0.08 (3.22)	0.56 (3.34)
Ages 80–89	26.3	30.8	0.17 (6.49)	0.64 (3.87)
Ages 90–99	27.3	54.9	0.26 (10.2)	0.66 (3.97)
Ages 100+	19.5	70.1	0.36 (13.9)	0.70 (4.20)
Women	57.3	42.2	0.24 (9.45)	0.69 (4.13)
Men	42.7	36.3	0.16 (6.18)	0.57 (3.41)
Non-Han ethnicity	6.6	43.7	0.18 (7.08)	0.67 (4.02)
Han ethnicity	93.4	39.3	0.21 (8.12)	0.63 (3.81)
No coresidence with family	16.7	35.7	0.18 (6.86)	0.67 (4.03)
Coresidence with family	83.3	40.4	0.21 (8.30)	0.63 (3.78)
Not currently married	69.1	48.2	0.25 (9.56)	0.67 (4.03)
Currently married	30.9	20.5	0.12 (4.69)	0.56 (3.36)
Not currently smoking	82.1	41.3	0.22 (8.68)	0.65 (3.88)
Currently smoking	17.9	32.3	0.13 (5.21)	0.60 (2.58)
Not doing regular exercise	72.8	44.6	0.24 (9.18)	0.67 (4.03)
Doing regular exercise	27.2	26.4	0.13 (5.07)	0.54 (3.26)

All numbers are unweighted. All numbers refer to the 2008/2009 wave, with exception for percent died, which refers to the period of 2008/2009-2011/2012

In order to see the percentage distributions of death, frailty, and socioeconomic vulnerability by age, in this table age is measured by age groups. In the regression analyses, age is measured by single year of age

^a^ The score of frailty index ranges from 0 (the healthiest) to 1 (the worst) with the number in parentheses indicating the average number of deficits ranging from 0 (the healthiest) to 39 (the worst)

^b^ The score of the socioeconomic vulnerability index ranges from 0 (the least vulnerable or the highest SES) to 1 (the most vulnerable or the lowest SES) with the number in parentheses indicating the average number of vulnerable socioeconomic conditions ranging from 0 (highest SES) to 6 (lowest SES)

Model I in Table 2 presents hazard ratios (HRs) for frailty and SES from a hazard model controlling for basic demographics, showing that both frailty index and SEVI were significant and independent predictors of mortality. A one percentage point increase in the frailty index was associated with an increased hazard ratio (HR) by 2.7 % (HR = 1.027, 95 % CI: 1.025–1.027); a one percentage point increase in SEVI was associated with an increased hazard ratio by 0.6 % (HR = 1.006, 95 % CI: 1.004–1.008). The HR of the interaction between SEVI and frailty index in Model II was less than 1 (HR = 0.983, 95%CI: 0.967–0.992, *p* < 0.001), and the result was similar when psychosocial covariates were controlled in Model III. This indicates that the increased mortality risk among frailer older adults was weaker in lower SES groups as compared to that in the higher SES groups.

**Table 2:** Mortality hazard ratios and 95 % CIs for frailty, socioeconomic vulnerability, and interactions, CLHLS 2008/2009-2011/2012

	Model I	Model II	Model III	Model IV	Model V	Model VI	Model VII	Model VIII
Main terms								
Frailty index (×100)	1.027*** (1.025–1.029)	1.038*** (1.032–1.044)	1.041*** (1.036–1.046)	1.032*** (1.025–1.039)	1.108*** (1.092–1.125)	1.105*** (1.088–1.122)	1.101*** (1.084–1.119)	1.099*** (1.081–1.117)
SEVI (×100)	1.006*** (1.004–1.008)	1.011*** (1.001–1.014)	1.012*** (1.009–1.015)	1.044*** (1.028–1.060)	1.008*** (1.005–1.011)	1.007*** (1.004–1.010)	1.033*** (1.017–1.049)	1.029*** (1.013–1.045)
Interaction terms								
SEVI*frailty index (×100)		0.983*** (0.967–0.992)	0.977*** (0.970–0.985)	0.993 (0.984–1.003)	0.991* (0.983–0.999)	0.994 (0.985–1.002)	0.996 (0.989–1.005)	0.998 (0.989–1.007)
SEVI*age				0.963*** (0.946–0.980)			0.973** (0.956–0.990)	0.975** (0.958–0.993)
Frailty index*age					0.928*** (0.913–0.943)	0.928*** (0.913–0.944)	0.930*** (0.915–0.946)	0.931*** (0.915–0.946)
Controls								
Age	1.055*** (1.052–1.058)	1.055*** (1.051–1.058)	1.054*** (1.050–1.058)	1.081*** (1.068–1.094)	1.074*** (1.069–1.080)	1.070*** (1.065–1.076)	1.094*** (1.080–1.107)	1.088*** (1.074–1.101)
Men (women)	1.510*** (1.421–1.605)	1.511*** (1.423–1.601)	1.563*** (1.0464–1.667)	1.501*** (1.413–1.594)	1.485*** (1.399–1.576)	1.535*** (1.439–1.638)	1.478*** (1.392–1.569)	1.526*** (1.430–1.628)
Han ethnicity (non-Han)	0.866** (0.784–0.956)	0.868** (0.785–0.957)	0.889* (0.805–0.982)	0.861** (0.780–0.951)	0.873** (0.791–0.964)	0.895* (0.810–0.988)	0.869** (0.787–0.960)	0.891* (0.806–0.984)
Coresidence with family (no)			1.096* (1.013–1.184)			1.090* (1.009–1.178)		1.090* (1.009–1.178)
Currently married (no)			0.769*** (0.705–0.838)			0.779*** (0.715–0.849)		0.786*** (0.721–0.856)
Currently smoking (no)			1.056 (0.973–1.146)			1.072 (0.987–1.162)		1.074 (0.990–1.18)
Doing regular exercise (no)			0.878*** (0.817–0.944)			0.863*** (0.802–0.928)		0.864*** (0.804–0.929)
Wald Chi square	4013.0***	4006.1***	3844.8***	3891.2***	3630.1***	3608.4***	3558.2***	3547.3***

Score of frailty index ranges from 0 (no deficits, the healthiest) to 1 (all deficits, the worst), while the score of the socioeconomic vulnerability index ranges from 0 (the least vulnerable) to 1 (the most vulnerable)

The reference category for each control is in parentheses

The total valid sample size in the analyses is 13,731

A model that includes the interaction between SEVI and age, main terms, and controls is similar to Model IV. A model that includes the interaction between frailty index and age, main terms and controls is similar to Model V. Therefore, these two models are not presented to save the space

*p <0.05, ^**^ *p* <0.01, ^***^ *p* <0.001

Our results also show that the weaker association between frailty and mortality in poorer SES groups disappeared once the interaction between socioeconomic vulnerability and age was controlled for (Model IV). Although the frailty*SEVI interaction was still significant when the interaction between frailty and age was controlled for in Model V, it was again not significant when psychosocial covariates were additionally controlled for in Model VI. These results imply that the interaction between frailty and SEVI could be explained by the interaction between socioeconomic vulnerability and age, the interaction between frailty index and age, and psychosocial factors.

The significant interactions between socioeconomic vulnerability and age and between frailty and age in Models IV to VI suggest that the increased mortality risk associated with socioeconomic vulnerability (Model IV) and frailty (Model V) were weakened with advanced age. These findings were not altered when all three two-way interactions and psychosocial covariates were included in the analysis (Models VII and VIII). This suggests that socioeconomic vulnerability and frailty are stronger determinants of mortality risk among the young-old population than among the old-old population.

## Discussion

Using a large nationally representative survey of mainland China, a developing country that has witnessed societal transition in the past few decades, we investigated the moderating role of SES in the linkage between frailty and mortality. One of the significant contributions of the present study to the existing literature is that frailty is less strongly associated with mortality among individuals with low SES than among individuals with high SES. There are several possible explanations for this relationship, including material, behavioral, and psychological pathways. First, people with high SES have more access to health-related innovation, information, and resources compared to those with lower SES [45, 46]. People with high SES could have good living and working environments that benefit health in both short and long terms [24, 47]. These benefits may help to maintain health or reduce deficits. Second, prior studies have shown that high SES is associated with healthier behaviors and lifestyles, which are protective factors for mortality and health deficits [31, 32, 45, 47]. Third, higher SES confers psychological advantages, such as higher levels of self-efficacy and coping abilities and lower psychological distress [32, 47–49]. Such advantages are likely absent among populations with lower SES. In addition, a high SES is an important factor that could preserve reserve capacities and mitigate the progression of functional limitation and health decline over time [50, 51]. Considering all these advantages of high SES, a frail person in the high SES group likely reflects a very poor condition. In that context, frailty in the low SES group might be more normative and less indicative of serious health problems compared to frailty in the high SES group. In sum, the moderating role of SES stems from the fact that SES provides additional resources and advantages that buffer the impact of frailty on mortality or low SES may introduce selection that weakens the effect of frailty on mortality at older ages.

Our findings are in line with studies from USA, UK, Brazil, and Sweden that found a stronger impact of self-rated health on mortality for the highest versus lowest educational or income group [33–35, 38]; however, our findings are inconsistent with other studies that found either no significant interactions between self-rated health, education, and income on mortality [39–41] or an opposite conclusion [36, 37]. One reason for these discrepancies is that a single measure of self-rated health does not necessarily reflect "actual health", although objective health conditions do affect self-rated health [52–54]. It is difficult to fully gauge how much of the socioeconomic differences in self-rated health are attributable to ‘true’ health differences and how much are due to differences in individuals' own norms and expectations that influence self-ratings [35]. By contrast, the frailty index that combines both objective and subjective components may be better able to capture the true level of overall health. Second, unlike many prior studies that focused on the general adult population, our study focuses on older adults. Associations between health conditions and mortality vary across age groups [52–54]. Third, provided that individuals with high education and high income likely have greater health literacy and/or access to health services that enable them to assess their own health more accurately, making self-rated health a better predictor of mortality risk in the high SES population [35], it is thus possible that the Chinese older adults rated their health less accurately than their counterparts in most Western countries due to their lower health literacy and education and per capita income [19, 55–57].

We also found that the weakening link between frailty and mortality among older adults with lower SES was diminished when the interactions of age with SES and frailty were taken into consideration. We further found that the significant predictive power of frailty on mortality is valid for all ages, yet the higher mortality risk associated with higher frailty index score was weakened in the older old compared to that in the younger old. Mortality risk of higher socioeconomic vulnerability was also weakened in the older old in comparison to their younger counterparts. These findings support some recent findings on a diminished effect of frailty on mortality in centenarians compared to other old age groups [19, 20]. We speculate that these age-as-leveler patterns may be attributable to the greater homogeneity in cumulated physical, psychological, and social deficits at older ages due to the increasing biological forces, or mortality selection that likely reduces the power of frailty index with age [20, 58, 59]. Overall, our findings imply that SES differentials in the linkage between frailty and mortality weakened with advanced age, yet the increased mortality associated with higher frailty persisted.

Some patterns less relevant to the central points of the present study are worth mentioning. First, the SEVI value increases with age, indicating that socioeconomic vulnerability of older people may be due to generational or cohort effects [60]. Second, compared to non-coresidence with family, coresidence with family was associated with a greater frailty index score and a higher mortality risk. This is mainly because many older adults who coreside with their children are in poor health and need to be cared for by the family members. This finding is in line with the literature [61–64].

One strength of the study is the application of the frailty index to construct a more robust indicator of health to better capture the multifaceted overall health condition. The cumulative deficit approach to frailty captures a relatively complete inventory of physical, cognitive, and psychological deficits that accumulate over the life course, rather than specific health outcomes of diseases or disabilities [2–8]. Another strength of the study is the construction of a cumulative SEVI. We followed the cumulative approach to a social vulnerability index proposed by Andrew and colleagues [11] to generate a single socioeconomic vulnerability index with several advantages, including its multidimensional construct that overcomes difficulties in modeling a large number of components [65, 66], better reflections of the actual association between SES and mortality [2–4], avoidance of less well defined measures that are readily applicable in younger people [11, 67], and inclusion of family level situation that reflects family members' financial interdependence [11], which is especially important in China where filial piety still prevails [68]. Other strengths of the present study include a nationally representative population-based dataset, a large sample of many very old adults who are frail yet understudied in the existing literature, and prospective cohort study. All these strengths allowed meaningful and robust estimates of risks of mortality associated with frailty, SES, age, and interactions. Undoubtedly, the SES differentials in the association between frailty and mortality require further investigations and more research from other elderly populations to shed light on this theme.

While emphasizing the advantages of the present study, we identify several avenues for future research. First, in constructing the frailty index, deficit items were not weighted. How to weight each of these items has been a challenge. Further research on this topic is clearly warranted. Second, our frailty index did not include some clinical, biomarker, and psychological resilience components that have been incorporated in different forms of frailty index [4, 69–71], although some studies have noted that omission of some items in constructing frailty index is less sensitive to its validity and reliability when the number of deficits reach a certain level [7, 43]. Third, variables used in constructing the socioeconomic vulnerability index may not be complete. In China, intergenerational transfer is still common, yet our socioeconomic vulnerability index did not include much information on it. Furthermore, similar to social vulnerability [11], our SEVI is a relatively new construct and the reliability coefficient of the index is not very high; more research is clearly warranted to investigate how to construct a more robust socioeconomic vulnerability index in Eastern countries as well as in Western societies. Undoubtedly, as our understanding of frailty and mortality and the moderating roles of SES and age increases, future studies will continue to refine measures of frailty and SES.

Because of the widespread use of frailty index in clinical, public health, and social science research, our research highlights some important theoretical and practical implications. SES differences in the linkage between frailty and mortality may help us understand health inequalities and the causes of those inequalities at old ages. Our findings imply that public health programs aimed at improving SES, reducing or eliminating socioeconomic disparities, and effectively promoting healthy longevity for older adults should start early in old age, or even earlier, and target poor and frail older adults for maximum impact [6, 17, 46]. A broad approach that develops nationwide programs or systems, such as a universal coverage of basic medical care services, compulsory education program, social welfare/social security, and subsidy programs to the poor and the frail populations, could be important influences on well-being and mortality in later life.

## Conclusions

Frailty has been widely studied as one of the crucial outcomes in monitoring public health responses to the challenges of population aging [46]. Using a large unique nationwide prospective dataset in China, this study found significant linkages between frailty and mortality and between socioeconomic vulnerability and mortality, and a stronger predictive power of frailty on mortality risk among individuals with higher SES than among individuals with lower SES. Greater mortality risk associated with lower SES and poorer health weakened with age. These findings could offer some insights into the role of SES on health disparities at older ages.

## Abbreviations

CLHLS, Chinese Longitudinal Healthy Longevity Survey; HR, hazard ratio; SES, socioeconomic status; SEVI, socioeconomic vulnerability index

## Figures and Tables

**Fig 1:**
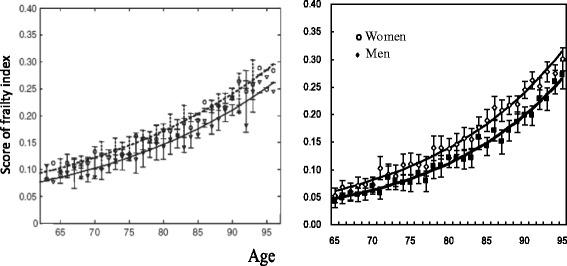
Comparison of age-specific distribution of frailty index between previous studies and the CLHLS in 2008/2009. *Left panel* obtained from Mitnitski et al. [72] with a permission. *Right panel*: authors’ own calculation from the CLHLS 2008/2009 to 2011/2012 wave

**Fig. 2:**
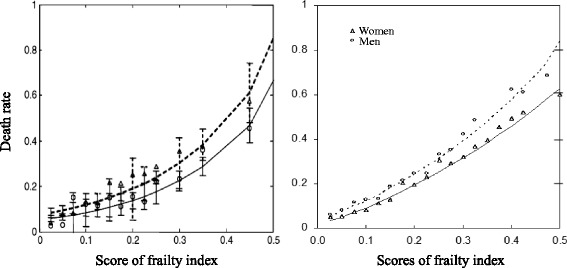
Comparison of mortality rate by frailty index score between previous studies and the CLHLS 2008/2009-2011/2012. *Left panel* obtained from Mitnitski et al. [72] with a permission. *Right panel*: authors’ own calculation from the CLHLS 2008/2009 to 2011/2012 wave

## Appendix 3

**Table 3:** Mortality hazard ratios for frailty, socioeconomic vulnerability, and interactions based on including those lost to follow-up, CLHLS 2008/2009-2011/2012

	Model I	Model II	Model III	Model IV	Model V	Model VI	Model VII	Model VIII
Main terms								
Frailty index (×100)	1.029***	1.043***	1.041***	1.037***	1.112***	1.110***	1.105***	1.104***
SEVI (×100)	1.006***	1.013***	1.011**	1.049**	1.010***	1.009***	1.040***	1.036***
Interaction terms								
SEVI*frailty index (×100)		0.980***	0.982***	0.989**	0.986***	0.988**	0.992*	0.993+
SEVI*age				0.960***			0.967***	0.969***
Frailty index*age					0.930***	0.930***	0.932***	0.932***
Controls								
Age	1.059***	1.058***	1.054***	1.087***	1.078***	1.074***	1.102***	1.096***
Men (women)	1.493***	1.494 ***	1.548***	1.483***	1.478***	1.522***	1.469***	1.510***
Han ethnicity (non-Han)	0.844***	0.847***	0.867**	0.842***	0.851**	0.871**	0.848**	0.867**
Coresidence with family (no)			1.106**			1.112**		1.132**
Currently married (no)			0.772***			0.788***		0.797***
Currently smoking (no)			1.043			1.064		1.068+
Doing regular exercise (no)			0.872***			0.866***		0.868***
N	16,563	16,563	16,563	16,563	16,563	16,563	16,563	16,563
Wald Chi square	5544.1***	5570.0***	5525.4***	5401.5***	4986.1***	4947.5***	4853.2***	4843.6***

Relative hazards were estimated based on inclusion of those lost to follow-up whose survival status and days survived from the interview in 2008 to the interview in 2011 were imputed. The imputation assumed that those who were lost to follow-up had the same survival status and survival length with those who were not lost to follow up given the same demographics, frailty index score, SEVI score, family/social support and health practice

Score of frailty index ranges from 0 (no deficits, the healthiest) to 1 (all deficits, the worst), while the score of the socioeconomic vulnerability index ranges from 0 (the least vulnerable) to 1 (the most vulnerable)

The reference category for each control is in parentheses

A model that includes the interaction between SEVI and age, main terms, and controls is similar to Model IV. A model that includes the interaction between frailty index and age, main terms and controls is similar to Model V. Therefore, these two models are not presented to save the space

+p <0.1, **p* <0.05, ^**^ *p* <0.01, ^***^ *p* <0.001
