# Erratum to: Socioeconomic status as a moderator between frailty and mortality at old ages

**DOI:** 10.1186/s12877-016-0330-2

**Published:** 2016-09-08

**Authors:** Danan Gu, Fang Yang, Jessica Sautter

**Affiliations:** 1 United Nations Population Division, Two UN Plaza, DC2-1910, New York, NY 20012 USA; 2 Department of Social Work, School of Sociology and Political Science, Shanghai University, Shanghai, China; 3 Department of Behavioral and Social Sciences, University of the Sciences, Philadelphia, USA

## Erratum

Unfortunately, the original version of this article [1] contained an error within the results section of the abstract. The sentence beginning with “We found that a one percentage point increase” read “....HR = 1.027, 95 % CI: 1.025–1.027…” but should have read “…HR = 1.027, 95 % CI: 1.025–1.029…” The upper limit of “1.027” was wrong, and was inconsistent with Table 2. This has been corrected in the original article.
